# Supplementary figures and images for: In vitro transcription accurately predicts lac repressor phenotype in vivo in Escherichia coli
Source: PeerJ. 2014 Jul 29;2:e498. doi: 10.7717/peerj.498 (PMC4121545; doi:10.7717/peerj.498)

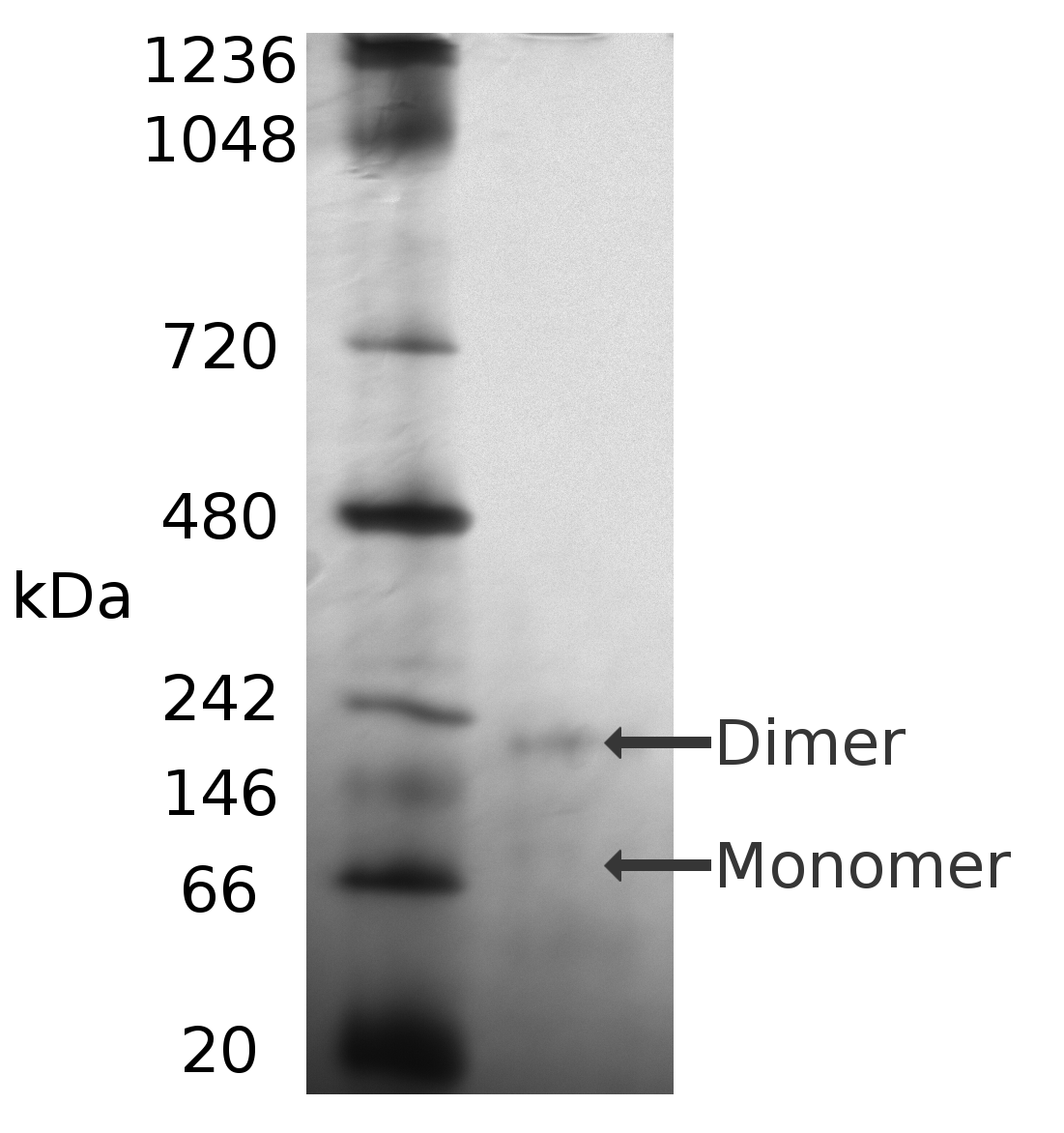

Supplement: Figure S1 — The purified Lac-mCherry protein was run on native gel electrophoresis. Expected band sizes for this protein are 65 kDa for monomer and 131 kDa for dimer. A single band is observed slightly higher than the 146 kDa marker which would indicate than nearly all of the Lac-mCherry protein is in the dimeric state (>95%). [file peerj-02-498-s001.png]

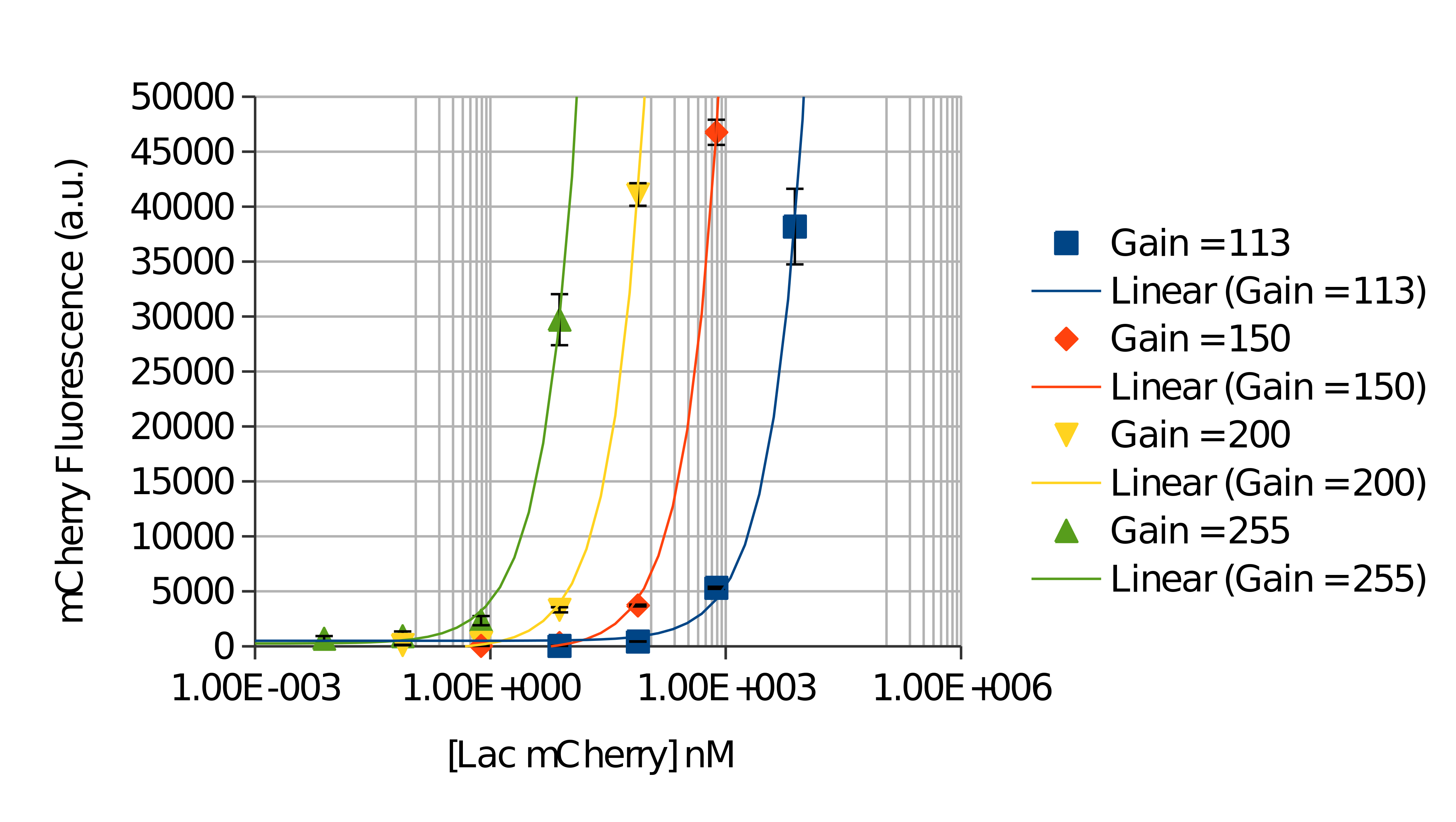

Supplement: Figure S2 — Purified Lac mCherry was diluted over 8 orders of magnitude and mCherry fluorescence was measured at various plate reader gains. The linear range of the plate reader was consistently accurate over four orders of magnitude for a given concentration range and gain. The concentrations measured in vivo corresponded with a gain of 255 for our instrument as described in the methods. [file peerj-02-498-s002.png]
